# Association Between Postoperative NSAID Use and Bleeding Following Transoral Robotic Surgery

**DOI:** 10.1002/ohn.70279

**Published:** 2026-04-30

**Authors:** Brayden Seliger, Jacob Beiriger, Milana Berry, Nilam Patel, Richard B. Cannon, Marcus M. Monroe, Hilary C. McCrary

**Affiliations:** ^1^ Department of Otolaryngology–Head and Neck Surgery University of Utah Salt Lake City Utah USA

**Keywords:** enhanced recovery after surgery, NSAIDs, pain management, postoperative hemorrhage, transoral robotic surgery

## Abstract

**Objective:**

To evaluate the safety and outcomes of NSAID use following transoral robotic surgery (TORS).

**Study Design:**

Retrospective cohort study using propensity score matching.

**Setting:**

Multi‐institutional database (TriNetX).

**Methods:**

Patients undergoing TORS were identified using ICD‐10 codes. Two propensity‐matched cohorts were compared: (1) patients receiving NSAIDs (ketorolac, celecoxib, ibuprofen) within 14 days postsurgery (n = 3639) versus controls (n = 3639) and (2) patients receiving ketorolac day‐of‐surgery (n = 1901) versus controls (n = 1901). Primary outcome was postoperative hemorrhage. Secondary outcomes included critical care admission, emergency department visit, and feeding device placement within 14 days.

**Results:**

Postoperative bleeding rates were similar between NSAID and control groups (*P* = .150). Patients treated with NSAIDs had lower rates of critical care admission (*P* < .001) and feeding tube placement (*P* < .001). Emergency department visits showed no significant difference (*P* = .813). Day‐of‐surgery ketorolac versus control showed no increased bleeding (*P* = .460). Ketorolac patients demonstrated significantly lower rates of critical care admission (*P* < .001) and feeding tube placement (*P* < .001), with no increase in emergency department visits (*P* = .312).

**Conclusion:**

NSAID administration following TORS was not associated with increased postoperative hemorrhage. NSAID use was associated with reduced critical care utilization and feeding tube requirements. These findings support the safety of NSAIDs in multimodal analgesia protocols for TORS patients.

Transoral robotic surgery (TORS) was introduced in 2005 and has fundamentally changed surgical management of oropharynx squamous cell carcinoma.^1^, ^2^ TORS provides minimally invasive access and surgical precision for oropharyngeal disease while minimizing adverse effects on speech and swallowing outcomes.^2^, ^3^, ^4^ TORS represents a major advancement in operative technique, but postoperative analgesia for these patients can be challenging.^5^ Pain following TORS is a driver of morbidity, delay in recovery, and prolonged hospitalization; optimal management strategy following TORS lacks standardization and evidence‐based protocols.^5^, ^6^

Controversy in postoperative analgesia is not without rationale; NSAID inhibition of platelet aggregation has historically raised concerns for postoperative hemorrhage.^7^ Bleeding after TORS can be catastrophic due to rapid airway compromise—the American Society of Clinical Oncology cites hemorrhage as the primary acute risk after TORS.^8^ Studies examining NSAID use after oropharyngeal procedures present conflicting results. A meta‐analysis of pediatric tonsillectomy patients found a significant increase in bleeding with ibuprofen.^9^ However, other studies report no clinically meaningful increase in hemorrhage, return to the OR for bleeding, or transfusion with perioperative NSAIDs (including ibuprofen, ketorolac, or COX‐2–selective agents).^10^ Bleeding risk appears to be driven by patient and procedural factors (perioperative antithrombotic therapy, prior radiation, tumor extent/subsite, and comorbidity) rather than NSAID exposure.^8^

The potential benefits of NSAIDs in TORS patients may extend beyond analgesia. Effective pain control facilitates earlier oral intake, reduced length of stay, and decreased feeding tube placement.^5^ In the age of emphasizing enhanced recovery after surgery (ERAS) protocols and reduction in opioid usage, demonstrating safety of NSAIDs in TORS is important for care optimization.^5^, ^11^ Despite the growing adoption of TORS, no large‐scale studies have specifically examined the safety of NSAID use in TORS. We present a large retrospective cohort using a multi‐institutional database that examines associations between NSAIDs and postoperative outcome in patients undergoing TORS. We hypothesized that NSAID use would not increase bleeding risk while potentially improving functional recovery outcomes.

## Methods

We used TriNetX US Collaborative Network, a database of de‐identified electronic health records from over 120 million patients in 66 healthcare organizations across the United States.

Patients were identified using a compilation of International Classification of Diseases, Tenth Revision (ICD‐10) and CPT (Current Procedural Terminology) codes to select patients with transoral robotic‐assisted surgery. Robotic surgery was identified with ICD‐10‐PCS (procedure) codes specific for robotic‐assisted surgery (see Supplemental Table S1, available online for a comprehensive list of codes used). Patients meeting the index criteria of surgery from July 1, 2016, to May 1, 2025, were included. In the first analysis, patients were divided into two cohorts: (1) patients with TORS, and ketorolac, celecoxib, or ibuprofen and (2) patients with TORS, and no ketorolac, celecoxib, or ibuprofen on or within 14 days of surgery. In the second analysis patients divided into 2 cohorts: (1) patients with TORS, and ketorolac and (2) patients with TORS, and no ketorolac on day of surgery. The index date was defined as the date of surgery, and outcome capture began day of surgery. Outcomes included postprocedural hemorrhage, critical care services, operatively placed gastrostomy tubes, and emergency department visits.

To reduce confounding, patients in both analyses were matched 1:1 with their control group using greedy nearest‐neighbor propensity score matching (PSM). Patients were matched on Age, Female, White, Black or African American, and Unknown Ethnicity. Matching was assessed using standardized mean differences (SMDs), with values <0.1 indicating acceptable balance. Statistics and PSM analysis were performed with TriNetX Analytics. All outcomes were assessed within 14 days of the index surgery date.

This study received exempt approval from the University of Utah Institutional Review Board (IRB 00200941). The data reviewed is a secondary analysis of existing data, does not involve intervention or interaction with human subjects, and is de‐identified per the de‐identification standard defined in Section §164.514(a) of the HIPAA Privacy Rule. The process by which the data is de‐identified is attested to through a formal determination by a qualified expert as defined in Section §164.514(b)(1) of the HIPAA Privacy Rule. This formal determination by a qualified expert refreshed on December 2020.

## Results

The initial search identified 10,410 patients with ICD‐10 codes for TORS procedures. In the first analysis comparing NSAID use within 14 days postsurgery, 3712 patients received NSAIDs (ketorolac, celecoxib, or ibuprofen) and 6709 did not. After 1:1 propensity score matching, each cohort contained 3,639 patients with well‐balanced baseline characteristics (all standardized mean differences <0.1). The mean age was 56.3 ± 15.6 years in the NSAID group and 56.9 ± 15.1 years in the control group. Demographics were similar between groups, with 68.0% versus 68.8% White patients, 8.5% versus 7.8% unknown ethnicity, 7.6% versus 6.7% Black or African American patients, and 42.8% versus 43.5% female patients (Table 1).

**Table 1:** Propensity Score Matching Results—Population Summary Before and After Propensity Matching Occurred

Study 1: NSAIDs 0‐14 days after surgery								
	Before matching				After matching			
Characteristic	NSAIDs (n = 3712)	No NSAIDs (n = 6709)	*P*‐value	SMD	NSAIDs (n = 3639)	No NSAIDs (n = 3639)	*P*‐value	SMD
Age at Index (mean ± SD)	55.7 ± 16.3	59.0 ± 14.2	<.001	0.22	56.3 ± 15.6	56.9 ± 15.1	.08	0.041
White	2505 (67.5%)	4794 (71.8%)	<.001	0.095	2475 (68.0%)	2502 (68.8%)	.496	0.016
Female	1630 (43.9%)	2048 (30.7%)	<.001	0.276	1558 (42.8%)	1584 (43.5%)	.538	0.014
Unknown Ethnicity	311 (8.4%)	696 (10.4%)	.001	0.07	309 (8.5%)	283 (7.8%)	.265	0.026
Black or African American	278 (7.5%)	548 (8.2%)	.192	0.027	277 (7.6%)	242 (6.7%)	.111	0.037

Study 2: Ketorolac Day 0 After Surgery								
Characteristic	Ketorolac (n = 1901)	No Ketorolac (n = 8311)	*P*‐value	SMD	Ketorolac (n = 1901)	No Ketorolac (n = 1901)	*P*‐value	SMD
Age at Index (mean ± SD)	55.7 ± 15.6	58.5 ± 15.1	<.001	0.178	55.7 ± 15.6	55.9 ± 15.4	.75	0.01
White	1211 (63.7%)	5889 (71.2%)	<.001	0.16	1211 (63.7%)	1224 (64.4%)	.66	0.014
Female	710 (37.3%)	2835 (34.3%)	.011	0.064	710 (37.3%)	704 (37.0%)	.84	0.007
Unknown Ethnicity	129 (6.8%)	912 (11.0%)	<.001	0.149	129 (6.8%)	116 (6.1%)	.391	0.028
Black or African American	142 (7.5%)	650 (7.9%)	.572	0.014	142 (7.5%)	137 (7.2%)	.756	0.01

In the second analysis examining day‐of‐surgery ketorolac administration, 1901 patients received ketorolac and 8311 did not. After propensity matching, 1901 patients remained in each group with balanced characteristics. The mean age was 55.7 ± 15.6 years in the ketorolac group and 55.9 ± 15.4 in the control group. Patient demographics included 63.7% versus 64.4% White patients, 7.5% versus 7.2% Black or African American patients, 6.8% versus 6.1% unknown ethnicity, and 37.3% versus 37.0% female patients (Table 1).

Postoperative hemorrhage rates, defined by ICD‐10 codes J95.830 and K91.840 or CPT code 42962, were similar between treatment and control groups in both analyses. In the 14‐day NSAID analysis, hemorrhage occurred in 55 patients (1.5%) in the NSAID group compared to 71 patients (2.0%) in the control group (RR 0.78, 95% CI 0.55‐1.10, *P* = .150). Day‐of‐surgery ketorolac also showed no increased bleeding risk, with hemorrhage rates of 1.6% (n = 30) versus 1.9% (n = 36) in controls (RR 0.83, 95% CI 0.52‐1.35, *P* = .456).

**Figure 1:**
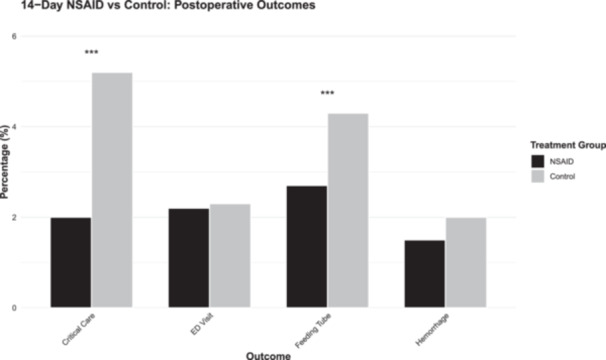
14‐Day NSAID versus control: postoperative outcomes. ***P < .001.

NSAID administration was associated with significantly reduced critical care rates. In the 14‐day analysis, 71 patients (2.0%) receiving NSAIDs required critical care services compared to 190 patients (5.2%) in the control group (RR 0.37, 95% CI 0.29‐0.49, *P* < .001). Day‐of‐surgery ketorolac demonstrated similar benefits, with critical care rates of 2.2% (n = 42) versus 4.7% (n = 90) (RR 0.47, 95% CI 0.33‐0.70, *P* < .001) (Figure 1).

**Figure 2:**
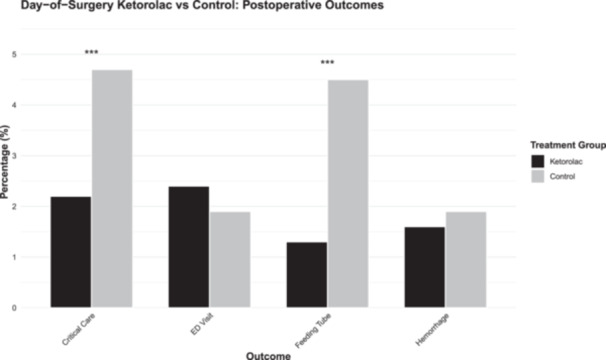
Day of surgery ketorolac versus control: postoperative outcomes. ***P < .001.

Operatively placed gastrostomy tubes were significantly lower in patients receiving NSAIDs. In the 14‐day analysis, 98 patients (2.7%) in the NSAID group required feeding tube placement compared to 158 patients (4.3%) in controls (RR 0.62, 95% CI 0.48‐0.80, *P* < .001). The effect was more pronounced with day‐of‐surgery ketorolac, with feeding tube placement rates of 1.3% (n = 25) versus 4.5% (n = 85) (RR 0.29, 95% CI 0.19‐0.46, *P* < .001) (Figure 2).

Emergency department utilization within 14 days showed no significant difference between groups. In the 14‐day NSAID analysis, 81 (2.2%) NSAID patients visited the emergency department compared to 84 patients (2.3%) in controls (RR 0.96, 95% CI 0.71‐1.30, *P* = .813). Similarly, day‐of‐surgery ketorolac showed no significant difference, with 45 (2.4%) ketorolac patients having emergency department visit versus 36 patients (1.9%) (RR 1.25, 95% CI 0.81‐1.93, *P* = .312).

All outcomes were assessed 0 to 14 days of the index surgery date (Table 2).

**Table 2:** Comparison of Outcomes Between NSAID and No NSAID Groups in Robotic Head and Neck Surgery

	Study 1: NSAIDs 0‐14 Days Post‐Surgery (n = 3639 per group)				Study 2: Ketorolac Day of Surgery (n = 1901 per group)			
Outcomes	NSAIDs n (%)	No NSAIDs n (%)	Risk Ratio (95% CI)	*P* value	Ketorolac n (%)	No Ketorolac n (%)	Risk Ratio (95% CI)	*P* value
Postprocedural hemorrhage	55 (1.5)	71 (2.0)	0.775 (0.546‐1.098)	.15	30 (1.6)	36 (1.9)	0.833 (0.515‐1.347)	.456
Critical care services	71 (2.0)	190 (5.2)	0.374 (0.286‐0.489)	<.001	42 (2.2)	90 (4.7)	0.467 (0.325‐0.669)	<.001
Gastrostomy tube placement	98 (2.7)	158 (4.3)	0.620 (0.484‐0.795)	<.001	25 (1.3)	85 (4.5)	0.294 (0.189‐0.457)	<.001
Readmission (ER visits)	81 (2.2)	84 (2.3)	0.964 (0.713‐1.304)	.813	45 (2.4)	36 (1.9)	1.250 (0.810‐1.929)	.312

## Discussion

Postoperative bleeding after oropharyngeal surgery can have devastating consequences due to the difficulty in controlling oropharyngeal hemorrhages, robust blood supply, and subsequent risk of airway obstruction.^8^, ^12^ In the climate of ERAS and opioid reduction, NSAIDs have emerged as a replacement and reduction agent for short‐ and long‐term opioids, but there is discord about whether they should be used in specific head and neck procedures.^5^, ^10^, ^13^, ^14^ Many surgeons are hesitant to include NSAIDs in recovery due to theoretical concerns of increased bleeding through cyclooxygenase blockade.^7^ Our results show that NSAIDs are not associated with increased postoperative hemorrhage in TORS patients. We also present decreased critical care utilization, feeding tube requirements, and comparable emergency department visits.

This study evaluates how NSAIDs influence postoperative outcomes in patients undergoing TORS. Postoperative hemostasis is critical in TORS but few studies look at associations between NSAID use and bleeding in TORS. A systematic review published in 2021 looked at 74 different studies in specialties ranging from otolaryngology to breast surgery found that there was no broad association between NSAID use and postoperative bleeding.^10^ Similarly, while TORS is fundamentally different from traditional tonsillectomy, it is reassuring that two systematic reviews of traditional tonsillectomy and NSAID use found no association with postoperative bleeding.^15^, ^16^ A 2013 review analyzed 36 studies and a 2025 review analyzed 26 studies, both included pediatric and adult populations, and neither found any association between NSAIDs and postoperative hemorrhage.^15^, ^16^

Van Abel et al looked at 216 patients undergoing TORS and found ibuprofen was not associated with postoperative hemorrhage.^11^ Sandelski et al looked at 71 patients undergoing TORS and found ketorolac was not significantly associated with increased bleeding, though they did raise concern of an insignificant increase in minor bleeds after leaving the hospital.^17^ The present study incorporates a much larger population, multiple institutions, and two groups: a ketorolac only group and a group including ketorolac, ibuprofen, and celecoxib. Our query includes patients from the TriNetX platform, which began its live electronic health record data in 2016 and includes historically imported data, through May 2025, whereas the Van Abel et al examined data from 2012‐2016. Van Abel et al reported a bleed rate as 5.6% with 1.4% returning to the operating room.^11^ We found similar rates ranging from 1.5% to 2.0% in our current study and both studies corresponded to the PATHOS trial which found 1.6% of TORS patients suffered serious hemorrhage.^18^ To investigate bleeds after discharge, emergency department visits were used as a surrogate for additional evidence of bleeding or other major adverse events after leaving the hospital and no significance was found.

The lack of increased bleeding with NSAIDs in TORS supports growing evidence that NSAIDs are likely not responsible for increased bleeding, but challenges traditional understanding of coagulation.^7^, ^10^ NSAIDs inhibit cyclooxygenase enzymes and impair platelet aggregation.^7^ However, this effect is reversible with non‐aspirin NSAIDs and may be clinically insignificant in the setting of normal coagulation.^7^ One consideration is that hemostasis may be favorable in TORS when compared to traditional tonsillectomy. The clear vision, high magnification, and focused lighting through the robot allows for precise cautery and better identification of bleeds, potentially creating more durable hemostasis.^4^ Furthermore, the dissection plane in TORS tonsillectomy is deep to the constrictors and within the parapharyngeal space compared to a traditional tonsillectomy in which the dissection plane is superficial to the constrictors. This approach may minimize violation of muscular perforators into the tonsil therefore lowering bleeding. Additionally, it is standard of care to ligate branches of the external carotid artery when performing TORS, which also drastically decreases the risks of a catastrophic bleed after surgery.^19^

While these mechanisms help explain NSAID safety, the dramatic reduction in critical care and gastrostomy tube requirements should not be overshadowed. Our results suggest that optimal pain control in the immediate postoperative period could have lasting effects on functional recovery. Pain is a significant contributor to both level of hospitalization and feeding tube dependence after oropharyngeal surgery.^6^, ^20^ By providing superior analgesia without sedating side effects, it is possibly allowing patients to return to a normal swallow faster.^6^, ^20^

These findings are relevant for clinical practice. While there are published head and neck surgery ERAS protocols that include ketorolac, many current ERAS protocols for TORS recovery do not include NSAIDs other than celecoxib.^5^, ^21^ Our data suggest these restrictions may be unnecessarily conservative, depriving patients of effective analgesia and potentially prolonging recovery. Implementation of NSAID‐inclusive protocols could reduce opioid requirements during a critical period when patients are at high risk for opioid‐related adverse events.^5^, ^13^ The timing or type of NSAID administration appears important, with day‐of‐surgery ketorolac showing the most pronounced benefits. There is evidence suggesting that pre‐emptive analgesia, is important to block pain pathways before chronic pain development.^22^ Incorporating ketorolac into TORS protocols from the day of surgery might optimize both short and long‐term outcomes.

There are limitations that warrant discussion. As a retrospective database study, causality cannot be established between NSAID use and improved outcomes. Selection bias could play a role in NSAID prescriptions. If surgeons preferentially avoided NSAIDs in patients perceived to be at higher bleeding risk such as those with larger tumors or prior radiation, it could skew results.^8^ The current TriNetX population is biased toward larger, more equipped institutions which could influence generalizability of our study. While TNM staging is available within the TriNetX database, it was only documented in ~2% of our population making it impossible to meaningfully assess its relationship with hemorrhage risk (Table 3). TriNetX lacks clinical details such as tumor size, resection extent, or specific surgical techniques that might influence bleeding risk. We also cannot determine NSAID dosing, duration, adherence, or whether the patient received the drug from prescription data alone. While PSM was used to balance demographics, residual confounding from unmeasured variables may have influenced the results. Our definition of bleeding relied on diagnostic codes and procedures for hemorrhage control, potentially missing minor bleeding events managed conservatively or those coded differently. Similarly, our classification of TORS is limited to ICD codes which could capture cases we did not intend to. The data for nasogastric feeding tube placement was limited by an absence of codes thought to be due to our reliance on CPT billing codes which can miss nasogastric tube placement during larger encompassing procedures. Gastrostomy tube placement being more reliable for because it is an independent procedure.

**Table 3:** Summary of Staging NSAIDs 0‐14 Days Post‐Surgery Not All Patients Had Staging Available

Stage	NSAIDs (%)	No NSAIDs n (%)	Total (%)
T0	<10	<10	<10
T1	17 (44.7)	61 (47.3%)	78 (46.7%)
T2	18 (47.4%)	58 (45.0%)	76 (45.5%)
T3	<10	<10	<10
T4	<10	<10	<10

Highest stage indicated in record used. Values <10 are rounded for anonymity.

Despite the limitations, this study's results are promising. TORS patients are receiving NSAIDs, and bleeding remains low. Validation is needed. Prospective studies aimed at confirming this data should include outcomes we measured while also including granular details missing from the database. We could not capture stage of disease, extent of resection, the degree of bleeding, timing or dosage of NSAIDs, pain scores, patient perceived quality of life, or more detailed reports of feeding tubes.

One consideration is whether specific patients, like those with more extensive resections or prior radiation, face a magnified risk for bleeding through NSAIDs and should continue to be approached more conservatively. Similarly, it is uncertain whether there are major benefits beyond the immediate recovery period.

## Conclusion

In this large, retrospective database study, NSAID use in TORS patients was not associated with increased postoperative hemorrhage and demonstrated significant associations with improved recovery outcomes. These findings challenge longstanding concerns for post‐TORS hemorrhage and support including NSAIDs in ERAS protocols for TORS patients. While prospective validation is still needed, this data is a promising lead on enhanced postoperative analgesia for TORS patients. As TORS continues to expand, finding new ways to safely improve recovery helps surgeons and patients alike.

## Author Contributions

**Brayden Seliger**, **BS**, conceptualization, methodology, investigation, writing (original draft), writing (review and editing); **Jacob Beiriger**, **BS**, conceptualization, methodology, writing (original draft), writing (review and editing); **Milana Berry**, **BS**, conceptualization, writing (review and editing); **Nilam Patel**, **MD**, conceptualization, methodology, writing (review and editing), supervision; **Richard B. Cannon**, **MD**, conceptualization, methodology, writing (review and editing), supervision; **Marcus M. Monroe**, **MD**, conceptualization, methodology, writing (review and editing), supervision; **Hilary C. McCrary**, **MD**, **MPH**, conceptualization, methodology, investigation, writing (original draft), writing (review and editing), supervision.

## Disclosures

### Competing interests

None.

### Funding source

None.

## Supporting information


**Supplemental Codes**‐ Electronic medical record codes used to capture patient cohorts and outcomes.

## Data Availability

All relevant data are included within the manuscript.
