# Supplementary material for: Association Between Postoperative NSAID Use and Bleeding Following Transoral Robotic Surgery
Source: Otolaryngol Head Neck Surg. 2026 Apr 30;175(2):402–8. doi: 10.1002/ohn.70279 (PMC13417960; doi:10.1002/ohn.70279)
Supplement: Supplementary file 1 — Supplemental Codes‐ Electronic medical record codes used to capture patient cohorts and outcomes. [file OHN-175-402-s001.docx]

**Supplemental Codes-** Electronic medical record codes used to capture patient cohorts and outcomes

| **Description** | **Type** | **Coding System** | **Code** |
| --- | --- | --- | --- |
| **Robotic Assisted Procedure of Head and Neck Region, Open Approach** | **Procedure** | **ICD-10-PCS** | **8E090CZ** |
| **Robotic Assisted Procedure of Head and Neck Region** | **Procedure** | **ICD-10-PCS** | **8E09XCZ** |
| **Robotic Assisted Procedure of Head and Neck Region, Via Natural or Artificial Opening** | **Procedure** | **ICD-10-PCS** | **8E097CZ** |
| **Malignant neoplasm of oropharynx** | **Diagnosis** | **ICD-10-CM** | **C10** |
| **Malignant neoplasm of base of tongue** | **Diagnosis** | **ICD-10-CM** | **C01** |
| **Malignant neoplasm of tonsil** | **Diagnosis** | **ICD-10-CM** | **C09** |
| **Ketorolac** | **Medication** | **RXNORM** | **35827** |
| **Celecoxib** | **Medication** | **RXNORM** | **140587** |
| **Ibuprofen** | **Medication** | **RXNORM** | **5640** |
| **Postprocedural hemorrhage of respiratory system organ** | **Diagnosis** | **ICD-10-CM** | **J95.830** |
| **Postprocedural hemorrhage of digestive system organ** | **Diagnosis** | **ICD-10-CM** | **K91.840** |
| **Control oropharyngeal hemorrhage; secondary surgical intervention** | **Procedure** | **CPT** | **42962** |
| **Critical Care Services** | **Procedure** | **CPT** | **1013729** |
| **Insertion of Feeding Device into Stomach, Open Approach** | **Procedure** | **ICD-10-PCS** | **0DH60UZ** |
| **Insertion of Feeding Device into Stomach, Percutaneous Approach** | **Procedure** | **ICD-10-PCS** | **0DH63UZ** |
| **Insertion of Feeding Device into Stomach, Via Natural or Artificial Opening Endoscopic** | **Procedure** | **ICD-10-PCS** | **0DH68UZ** |
| **ER visit - minimal care** | **Procedure** | **CPT** | **99281** |
| **ER visit - straightforward decision making** | **Procedure** | **CPT** | **99282** |
| **ER visit - low-level decision making** | **Procedure** | **CPT** | **99283** |
| **ER visit - moderate-level decision making** | **Procedure** | **CPT** | **99284** |
| **Age at Index** | **Characteristic** | **Demographic** |  |
| **Female Sex** | **Characteristic** | **Demographic** | **F** |
| **White Race** | **Characteristic** | **Demographic** | **2106-3** |
| **Black or African American Race** | **Characteristic** | **Demographic** | **2054-5** |
| **Unknown Ethnicity** | **Characteristic** | **Demographic** | **UN** |
